# Urinary Microbiome: Yin and Yang of the Urinary Tract

**DOI:** 10.3389/fcimb.2021.617002

**Published:** 2021-05-18

**Authors:** Virginia Perez-Carrasco, Ana Soriano-Lerma, Miguel Soriano, José Gutiérrez-Fernández, Jose A. Garcia-Salcedo

**Affiliations:** ^1^ GENYO, Centre for Genomics and Oncological Research, Pfizer, University of Granada, Granada, Spain; ^2^ Microbiology Unit, University Hospital Virgen de las Nieves, Biosanitary Research Institute (IBS.Granada), Granada, Spain; ^3^ Department of Physiology, Faculty of Pharmacy, Institute of Nutrition and Food Technology “Jose’ Mataix”, University of Granada, Granada, Spain; ^4^ Center for Intensive Mediterranean Agrosystems and Agri-food Biotechnology (CIAMBITAL), University of Almeria, Almeria, Spain

**Keywords:** microbiome, urinary tract, infection, disease, health, sequencing

## Abstract

The application of next generation sequencing techniques has allowed the characterization of the urinary tract microbiome and has led to the rejection of the pre-established concept of sterility in the urinary bladder. Not only have microbial communities in the urinary tract been implicated in the maintenance of health but alterations in their composition have also been associated with different urinary pathologies, such as urinary tract infections (UTI). Therefore, the study of the urinary microbiome in healthy individuals, as well as its involvement in disease through the proliferation of opportunistic pathogens, could open a potential field of study, leading to new insights into prevention, diagnosis and treatment strategies for urinary pathologies. In this review we present an overview of the current state of knowledge about the urinary microbiome in health and disease, as well as its involvement in the development of new therapeutic strategies.

## Introduction

The human body is colonized by at least the same number of microorganisms than somatic cells. In fact, the estimated ratio of bacteria to human cells is 1.3:1, considering nucleated and enucleated cells (Sender et al., 2016).

The term ‘microbiota’ is used to refer to the group of microorganisms associated to a specific biologic niche (Banerjee and Robertson, 2019). The human microbiota is composed of 500-1000 bacterial species, with genomes containing thousands of genes, which offers a much greater diversity and genetic versatility than the human genome (Locey and Lennon, 2016). Moreover, many other microorganisms in the human body, such as protozoa, fungus, archaea and virus, are also part of the human microbiota.

There is a consensus regarding the beneficial role of these microorganisms in maintaining the homeostasis in the human body. The microbiota carries out important protective functions against pathogens through the formation of a physical barrier and contributes to the development of the immune system (Wang B. et al., 2017).

Alteration in the microbiota has been associated with the development of several pathologies, such as obesity (Riva et al., 2017), inflammatory bowel disease (Walters et al., 2014), ulcerative colitis (James et al., 2015), Crohn disease (Gutin et al., 2019), non-alcoholic fatty liver disease (Ma et al., 2017), insulin resistance syndrome (Saad et al., 2016), type II diabetes mellitus (Sikalidis and Maykish, 2020), Alzheimer’s disease (Jiang et al., 2017), atopic eczema (Abrahamsson et al., 2012), allergies (Tanaka et al., 2017) or asthma (Wu et al., 2016), among others. Therefore, gaining insight into the microbial composition of the human body and its alteration in pathological conditions might favor the development of new prophylactic, diagnostic and therapeutic strategies.

Similarly, the term ‘microbiome’ refers to the group of microbial genomes in a specific environment (Cimadamore et al., 2019). The great importance of microbial communities in the human body promoted the study and characterization of the microbiome in different human healthy body environments. The Human Microbiome Project has analyzed the composition of bacterial communities in several human body niches such as the oral cavity, skin, gastrointestinal tract or vagina, and its role in health and disease (Huttenhower et al., 2012). Initially, the urinary bladder was not included in the study because urine was traditionally considered sterile due to the assumption of pathogenicity for all bacteria (Zasloff, 2007). However, the development of high-throughput DNA sequencing techniques has led to the identification of the commensal microbial community in the urinary tract (Wolfe and Brubaker, 2015).

In this review, the current state of knowledge in relation to the urinary microbiome, or urobiome, is presented, with a focus on its importance for health maintenance and its role in the development of disease.

## The Evolution of Urinary Microbiome Analysis: Methodology and Limitations

**Figure 1:**
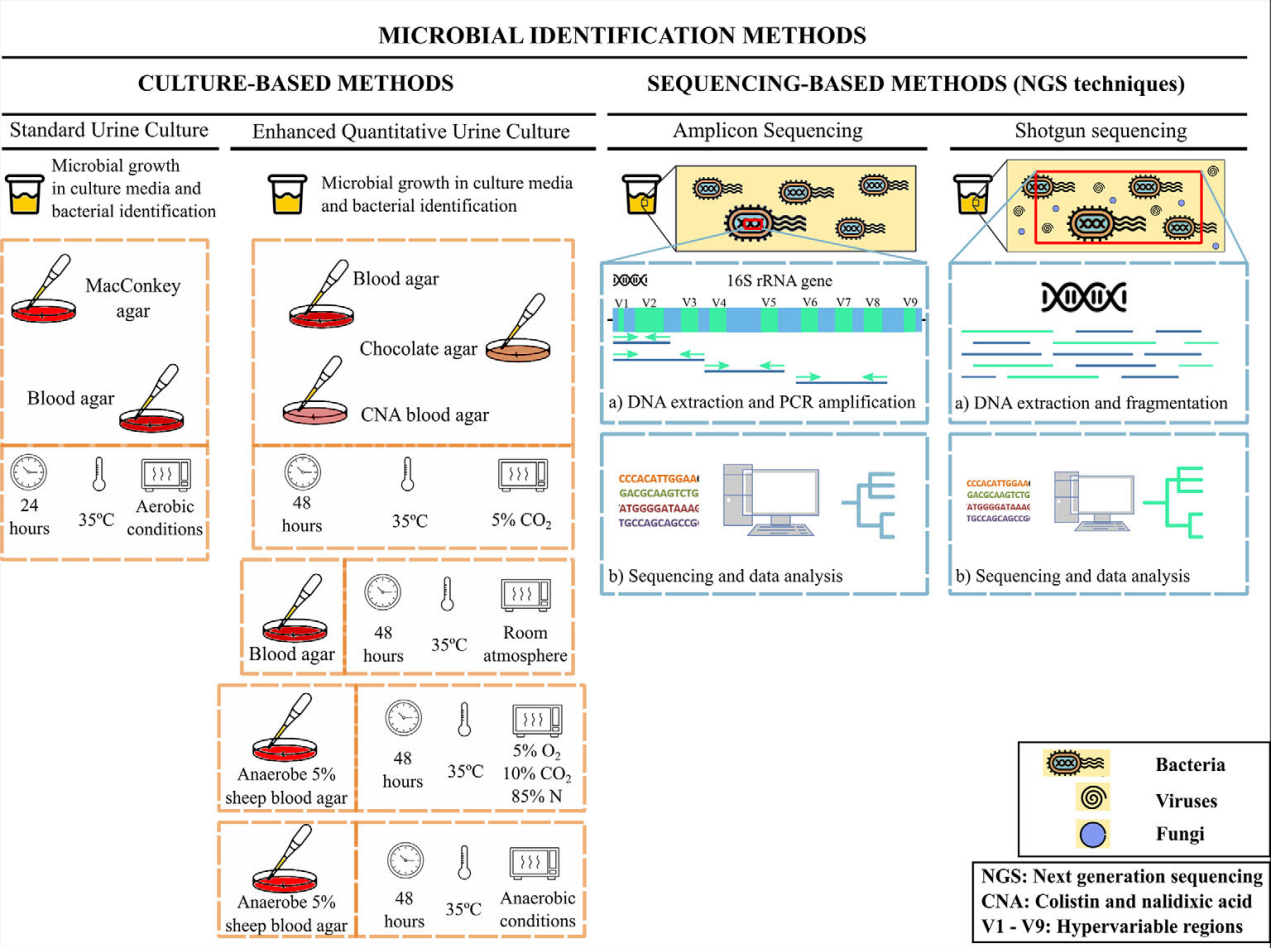
Microbial identification methods for urine samples. Bacteria in urine samples can be identified by different methods: culture-based methods (standard urine culture or enhanced quantitative urine culture (EQUC)) or sequencing-based methods (amplicon sequencing or shotgun sequencing).

Traditionally, the detection of microorganisms in the urinary tract was based on standard urine cultures (**Figure 1**), implemented in clinical microbiology laboratories. These methods only allow the detection of a limited number of microorganisms, mainly aerobic and fast-growing bacteria, such as *Escherichia coli* (García and Isenberg, 2010; Wolfe and Brubaker, 2015). Anaerobic microorganisms characterized by slow growth or bacteria with complex nutrient needs are, however, not detected using this approach.

The metagenomic analysis using Next Generation Sequencing (NGS) has facilitated the quantitative characterization of microbiomes, providing information on microbial populations and helping to discover uncultured microbes (Wolfe and Brubaker, 2015; Ishihara et al., 2020). Two approaches could be taken as far as NGS techniques are concerned: amplicon sequencing and shotgun sequencing (**Figure 1**). The first one consists of PCR-based analysis focused on marker genes, such as 16S rRNA subunit, with nine hypervariable regions (V1-V9) determining the evolutionary distance between different bacterial species and highly conserved interregional sequences for primer design (Wolfe and Brubaker, 2015; Aguilera-Arreola et al., 2016). The second one allows to sequence whole microbiome in a wide variety of samples (Jovel et al., 2016) including non-bacterial components such as viruses or fungi (Moustafa et al., 2018). NGS has enabled the identification of commensal and pathogenic species as well as identify new emerging uropathogens (Fouts et al., 2012; Lewis et al., 2013; Nienhouse et al., 2014).

However, one limitation of DNA sequencing-based methods is its inability to show the viability of the identified bacteria. For this reason, urine culture protocols have been improved aiming for broadening the range of potentially detectable bacteria. The use of the enhanced quantitative urine culture (EQUC) protocol is recommended to study the viability of bacteria in urine (**Figure 1**) (Price et al., 2016). This protocol consists of plating a urine sample in a set of media including blood agar plate, chocolate and colistin-nalidixic acid agars and incubating them under aerobic or anaerobic conditions at 35°C for 48 hours (Price et al., 2020). A greater diversity of bacteria can be cultured following this procedure, but if used as diagnostic tools, their results must be correctly interpreted since the identified microorganisms may appear in both patients and healthy individuals (Coorevits et al., 2017). Therefore, a combination of 16S rRNA gene sequencing along with EQUC would provide a reliable characterization of microbial communities in the urinary tract, since culture-based approaches would allow an approximate bacterial quantification and would therefore be useful as clinically relevant indicators.

**Figure 2:**
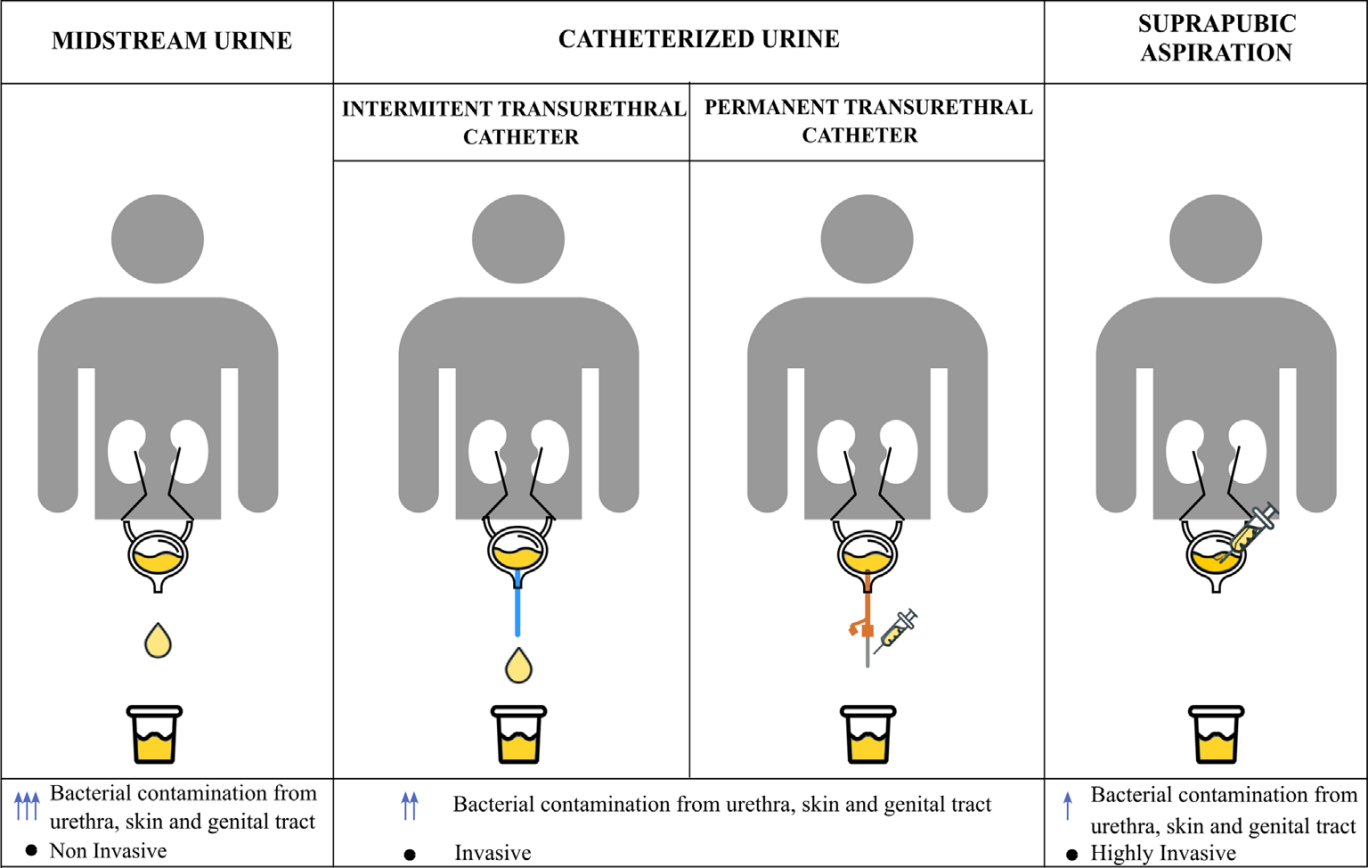
Collection methods for urine samples. Urine samples can be collected by different methods: collection of spontaneous midstream urine, catheterization with an intermittent or permanent catheter or suprapubic aspiration from the bladder. Differences between these methods lie in the grade of possible bacterial contamination from other areas such as the urethra, the skin or the genital apparatus, and the grade of invasion in each technique. The number of arrows indicates the grade of contamination depending on the collection method used.

Another relevant problem in urobiome studies is the choice of specific methods for sample collection, since those may affect the obtained results. The main current methods for urine sample collection are shown in **Figure 2**.

Many studies use midstream urine samples or spontaneously voided urine, being the its first milliliters discarded as they may be contaminated with bacteria from the skin (Lewis et al., 2013; Gottschick et al., 2017). Not being a sterile collection method, midstream urine samples may contain microbial contamination with bacteria from the uroepithelium, periurethra or the genital tract, thus misleading the proper characterization of the urinary bladder microbiome in favor of the urogenital microbiota (Fouts et al., 2012; Modena et al., 2017). Not even the joint use of funnels for urine collection and silver antimicrobial wipes have prevented sample contamination (Lough et al., 2019). This sample collection method presents issues related to the different sources of contamination in men and women, due to obvious anatomic differences. In women, the main source of contamination during midstream urine sample collection is the vulvovaginal tissue and it is not easily avoidable (Wolfe et al., 2012). The risk of contamination in midstream urine samples in men is lower, but there is also a chance of introducing microbial load from nearby tissues, such as urethra, is present (Kartens et al., 2018).

Another widely used method to collect urine samples consists of using transurethral catheters. Both straight intermittent and permanent transurethral catheters reduce the contamination compared to midstream urine sample. However, this is a more invasive technique and urethral bacteria might still contaminate samples during catheter insertion (Akmal-Hasan et al., 2014), especially in the case of permanent catheters. Hence, the best option for the collection of urine samples in studies regarding the urinary microbiome is suprapubic aspiration, since it is performed directly from the bladder avoiding the contact with other areas. Thus, data regarding the composition of the urinary bladder microbiome using this method are more accurate than those obtained through other routines (Eliacik et al., 2016). However, this is the most invasive, painful and complicated collection method (Badiee et al., 2014); a comparative study of microbial communities in urine obtained either with suprapubic aspiration or transurethral catheter has shown that the outcome is very similar regardless of the collection method, which makes transurethral catheterized samples widely accepted for the study of the urinary bladder microbiome (Wolfe et al., 2012).

A recent study has set the benchmark for the storage and collection conditions of urine when assessing the female urinary microbiome. The authors conclude that shorter storage times (periods not exceeding four days) and colder temperatures (below 4°C) are the most favorable conditions before long-term storage at -80°C; the reproducibility significantly improved when DNA preservatives (such as Assay Assure ^®^) were added to the sample (Jung et al., 2019).

The low biomass of the urinary microbiota (<10^5^ colony-forming units per milliliter, approximately) (Pearce et al., 2014) and its proximity to other bacterial niches with higher microbial biomass, such as the vagina or the gut, makes it necessary to take extreme precautions to avoid the introduction of contamination during sample collection, processing and data analysis (Kartens et al., 2018). Contaminant DNA is an important issue in microbiome studies, especially in low biomass communities where they might become more evident and contaminant taxa might be overrepresented. These contaminations can derive from laboratory environments, manipulation, reagents and amplicons or DNA from other samples (cross-contamination). For this reason, adding DNA extraction blanks and no-template controls is essential in microbiome studies (Eisenhofer et al., 2019).

## Urinary Microbiome or Urobiome

It is worth bearing in mind that the term “urinary microbiota”, refers to the microbial community in bladder-obtained urine, and its full characterization is still under development (Wolfe and Brubaker, 2015; Pohl et al., 2020). The number of reports on the human urobiome is very limited and most of these studies are focused on characterizing microbial communities in either women or men. In addition, due to the low number of cases included in these studies, and other variables such as gender, race or geographical distribution, it is still early to accurately define the composition of the urinary microbiome in the whole population and in specific subgroups, such as individuals suffering systemic diseases.

In general, the microbiota in urine is less abundant and less diverse than the microbiota in other sites of the body, such as the gut. For instance, the female urinary microbiota is estimated to contain 10^4^ – 10^5^ colony-forming unit (CFU)/ml in comparison with 10^12^ CFU/g in feces (Pearce et al., 2014).

Catheterized-urine used in most urinary microbiome studies provides us with an overview of the microbial community present in the urinary bladder. However, it is possible that some bladder mucosa-associated bacteria are not detected in these type of urine samples. For its detection it would be necessary to take biopsies or tissue samples. In fact, a recent study compared the urinary microbiota from catheterized-urine samples with the bladder mucosa-associated microbiota using tissue samples in patients suffering bladder cancer. Important differences in some taxa were found in this study, which suggest that the bladder tissue microbiota and the urinary microbiota might differ to some extent (Mansour et al., 2020).

**Figure 3:**
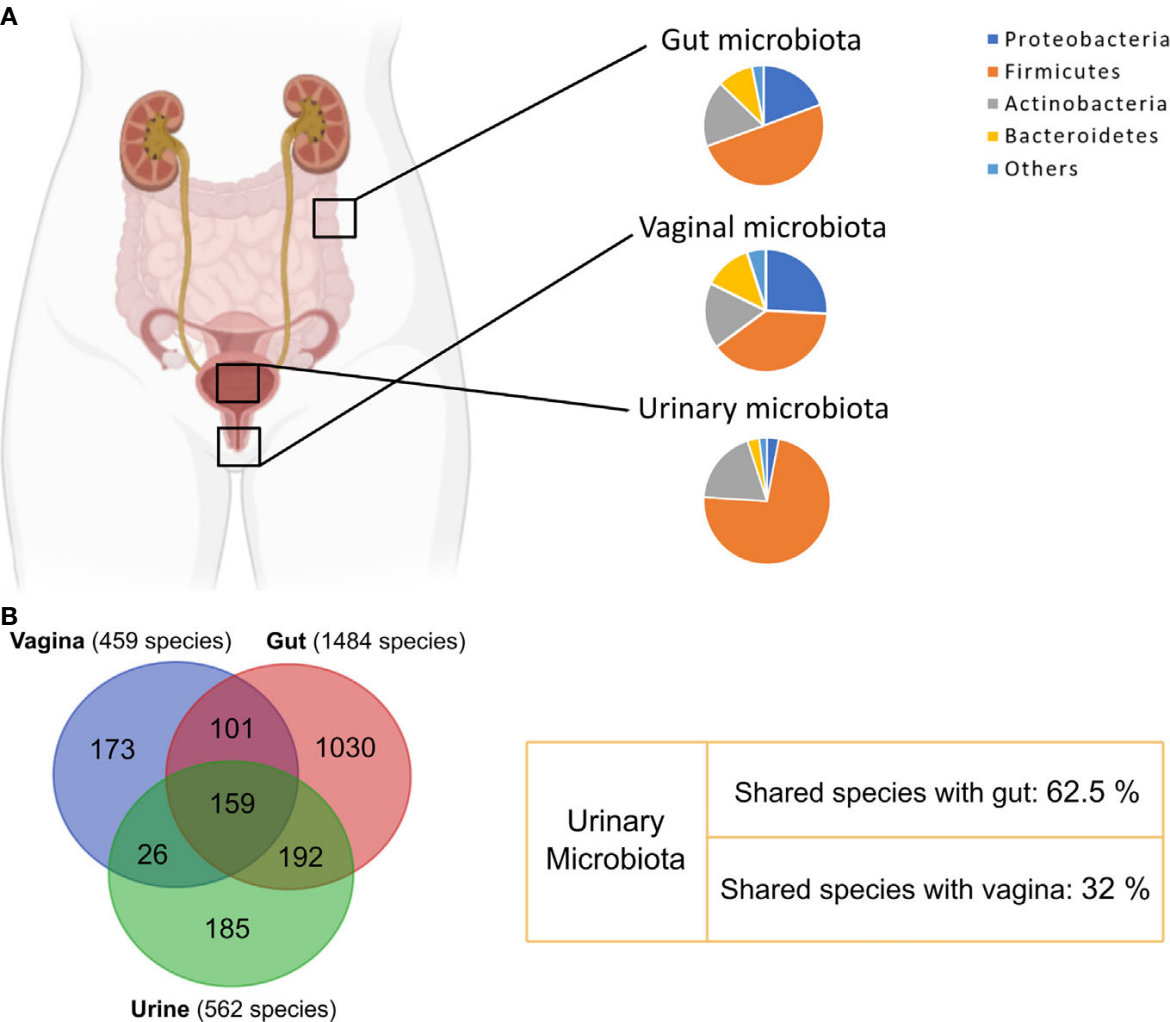
Comparison between urinary, vaginal and gut bacterial communities. **(A)** Phyla relative abundance in urinary (Modena et al., 2017), vaginal (Diop et al., 2019) and gut microbiota (Morand et al., 2019). **(B)** Venn diagram showing overlapping species between urinary (Morand et al., 2019), gut (Morand et al., 2019) and vaginal (Diop et al., 2019) microbiota.

The 16S rRNA sequencing-based characterization of the urinary microbiome, or urobiome, is similar for men and women at the phylum level. In both genders, the majority of bacteria belong to the Firmicutes phylum (65% in males *vs* 73% in females). The remainder phyla, accounting for up to 97% considering Firmicutes, are Actinobacteria (15% males *vs* 19% females), Bacteroidetes (10% males *vs* 3% females) and Proteobacteria (8% males *vs* 3% females) (Modena et al., 2017) (**Figure 3A**).

Most genera are shared in the urinary microbiome of healthy men and women. Common genera are *Prevotella, Escherichia, Enterococcus, Streptococcus* or *Citrobacter* among others, while genus *Pseudomonas* has only been described in men (Modena et al., 2017). Overall, the main difference in the composition of the urobiome between genders is found in the abundance of some genera such as *Corynebacterium* and *Streptococcus*, more abundant in men, or *Lactobacillus*, more abundant in women (Fouts et al., 2012; Modena et al., 2017). A general description of the main bacterial genera identified in the healthy female and male urinary microbiota is included in **Table 1**.

**Table 1:** Summary of the main genera identified in the healthy female and male urinary microbiota.

Reference	Sample collection method	Sample size (n)	Study technique	Main findings	
				**FEMALE URINARY MICROBIOTA**	**MALE URINARY MICROBIOTA**
Lewis et al., 2013	Midstream urine	10 ♀	16S rRNA sequencing, V1-V3 regions (FLX-titanium amplicon pyrosequencing)	70 genera identified: *Actinobaculum, Actinomyces, Aerococcus, Anaerococcus, Anaerosphaera, Anaerovorax, Arcanobacterium, Arthrobacter, Atopobium, Azospira, Brevibacterium, Brooklawnia, Butyricicoccus, Campylobacter, Catonella, Caulobacter, Coriobacterium, Corynebacterium, Dialister, Enterobacter, Enterocococcus, Facklamia, Fastidiosipila, Finegoldia, Flavonifractor, Friedmanniella, Fusobacterium, Gallicola, Gardnerella, Gulosibacter, Helcococcus, Howardella, incertae_sedis, Jonquetella, Lachnospiracea_, Lactobacillus, Methylovirgula, Microvirgula, Mobiluncus, Modestobacter, Murdochiella, Negativicoccus, Neisseria, Oligella, Paraprevotella, Parvimonas, Pelomonas, Peptococcus, Peptoniphilus, Peptostreptococcus, Porphyromonas, Prevotella, Propionimicrobium, Proteiniphilum, Rhodococcus, Rhodopila, Saccharofermentans, Sneathia, Soehngenia, Sporanaerobacter, Staphylococcus, Stenotrophomonas, Streptococcus, Streptophyta, Sutterella, Tepidimonas, Tessaracoccus, Thermoleophilum, TM7_genera_, Varibaculum*	46 genera identified: *Actinobaculum, Aerococcus, Aminobacterium, Anaerococcus, Anaerophaga, Anaerosphaera, Anaerotruncus, Atopobium, Atopostipes, Azospira, Butyricicoccus, Campylobacter, Catonella, Corynebacterium, Dialister, Eubacterium, Filifactor, Finegoldia, Fusobacterium, Gardnerella, Gemella, Gordonibacter, Kocuria, Lactobacillus, Lactonifactor, Marixanthomonas, Megasphaera, Microvirgula, Mobiluncus, Murdochiella, Mycoplasma, Parvimonas, Peptococcus, Peptoniphilu, Peptostreptococcus, Porphyromonas, Prevotella, Proteiniphilum, Pseudomonas, Pseudoramibacter, Rikenella, Saccharofermentans, Sediminitomix, Sneathia, Soehngenia, Staphylococcus*
		6 ♂			
Kogan et al., 2015	Midstream urine	24 ♀	Set of culture media and biochemical tests for bacterial identification	*Lactobacillus*, Coagulase-negative Staphylococci*, Peptococcus, Corynebacterium, Propionibacterium, Eubacterium, Peptostreptococcus, Candida, Bacteroides, Bacillus, Veillonella*, Enterobacteriaceae*, Staphylococcus aureus, Enterococcus, Micrococcus, Prevotella, Actinomyces, Streptococcus*	Coagulase-negative Staphylococci, *Eubacterium, Corynebacterium, Peptostreptococcus, Enterococcus, Bacteroides, Peptococcus, Megasphaera, Mobiluncus, Enterobacteriaceae, S. aureus, Propionibacterium, Veillonella, Fusobacterium*
		28 ♂			
Modena et al., 2017	Midstream urine	10 ♀	16S rRNA sequencing, V2-V4-V8 and V3-V6-V7-V9 regions, (Ion Torrent)	5 most abundant genera*: Lactobacillus, Corynebacterium, Gardnerella, Prevotella, Bacillus*	5 most abundant genera*: Streptococcus, Lactobacillus, Prevotella, Corynebacterium, Pseudomonas*
		10 ♂			
Gottschick et al., 2017	Midstream urine	49 ♀	16S rRNA sequencing, V1-V2 regions (Illumina)	10 urotypes identified. Predominant species in each urotype, respectively: *Prevotella amnii, Gardnerella vaginalis, Atopobium vaginae, Lactobacillus iners, Shigella sonnei, Escherichia coli, Enterococcus faecalis, Streptococcus agalacticie, Citrobacter murliniae, Lactobacillus crispatus*	6 urotypes identified. Predominant species in each urotype, respectively: *Prevotella amnii, Sneathia amnii, Shigella sonnei, Enterococcus faecalis, Streptococcus agalacticie, Citrobacter murliniae*
		31 ♂			
Fouts et al., 2012	Midstream or transurethral catheterized urine	15 ♀	16S rRNA sequencing, V1-V3 regions pyrosequencing (454 Roche)	5 most abundant genera*: Lactobacillus, Corynebacterium, Staphylococcus, Streptococcus, Prevotella*	5 most abundant genera: *Corynebacterium, Staphylococcus, Streptococcus, Lactobacillus, Veillonella*
		10 ♂			
Pearce et al., 2014	Transurethral catheterized urine	25 ♀	Enhanced Quantitative Urine Culture and 16S rRNA, V4 region (Illumina)	Main genera identified: *Lactobacillus, Staphylococcus, Corynebacterium, Enterobacteriaceae, Finegoldia, Streptococcus, Anaerococcus, Peptoniphilus, Varibaculum, Prevotella, Aerococcus, Gardnerella, Actinobaculum, Atopobium, Bifidobacterium, Alloscardovia*	
Thomas-White et al., 2018b	Transurethral catheterized urine	77 ♀	Standard urine culture, Enhanced Quantitative Urine Culture and whole genome sequencing (Illumina)	Main species identified: *Staphylococcus epidermidis, Micrococcus luteus, Lactobacillus gasseri, Escherichia coli, Streptococcus oralis, Neisseria perflava, Lactobacillus crispatus, Lactobacillus jensenii, Gardnerella vaginalis, Rothia mucilaginosa, Lactobacillus delbrueckii, Lactobacillus rhamnosus, Bacillus infantis, Actinomyces odontolyticus, Bacillus idriensis, Corynebacterium amycolatum, Streptococcus anginosus, Streptococcus agalactiae, Gordonia terrae, Staphylococcus warneri, Lactobacillus iners, Streptococcus mitis, Bifidobacterium bifidum, Streptococcus gordonii, Aerococcus urinae, Actinomyces neuii, Enterococcus faecalis, Streptococcus salivarius, Streptococcus sanguinis, Corynebacterium aurimucosum, Actinomyces naeslundii, Streptococcus equinus, Alloscardovia omnicolens, Corynebacterium tuscaniense, Bifidobacterium longum*	
Komesu et al., 2020	Transurethral catheterized urine	84 ♀	16S rRNA sequencing, V1-V3 regions (Illumina)	Main genera identified: *Lactobacillus, Streptococcus, Tepidimonas, Prevotella, Flavobacterium, Escherichia, Ureaplasma, Shuttleworthia, Aerococcus, Gardnerella, Veillonella, Bacteroides, Enterobacter, Acidovorax, Sneathia, Clostridium, Fusobacterium, Sphingobium, Proteus, Trabulsiella*	
Price et al., 2020	Transurethral catheterized urine	224 ♀	Enhanced urine culture and 16S rRNA sequencing, V4 region (Illumina)	4 predominant urotypes identified: *Lactobacillus, Gardnerella, Streptococcus* and *Escherichia* urotypes.	
				Other genera also identified: *Aerococcus, Alloscardovia, Anaerococcus, Bifidobacterium, Corynebacterium, Enterococcus, Finegoldia, Klebsiella, Prevotella, Staphylococcus*	

Some specific adaptations of the urinary microbiota to colonize the urinary tract have been described. Similar to the uropathogenic *Escherichia coli* (Sokurenko et al., 1998), some members of the urinary microbial community could use type 1 fimbriae to bind to specific urothelial proteins, uroplakins, using them as binding sites. Other factors such as pH, oxygen tension or nutrient availability have been described as key determinants; changes in oxygen tension have been associated with alterations in the urinary microbiota (Shannon et al., 2019). In addition, some members of the urinary microbial community, such as *Lactobacillus* or *Streptococcus*, have glycosaminoglycan-degrading enzymes, being able to metabolize the urothelium layer and favoring the availability of sugars (Kawai et al., 2018).

Most of the urobiome studies have been focused on bacteria although the presence of fungi, viruses and archaea in urobiome has also been described. The urinary fungal community has not been well characterized, especially in healthy individuals. The presence of *Candida* spp. has been reported in catheterized urine samples from healthy individuals (Pearce et al., 2014). Some preliminary data from a more recent study show the presence of a urinary fungal community formed mainly by individuals of Dothiodeomycetes, Saccharomycetes (where *Candida* belongs), Eurotiomycetes, Exobasidiomycetes and Microbotryomycetes classes (Ackerman and Underhill, 2017). However, the use of midstream urine samples in this analysis may have skewed the data due to possible contaminations from nearby tissues, such as vagina, where the presence of *Candida* has been widely documented (Drell et al., 2013). To date, only one species of archaea has been reported to be associated with disease in the urinary microbiome (Grine et al., 2019). On the other hand, the viral community in the urinary tract is mainly composed of bacteriophages, although some eukaryotic viruses have also been described. The urinary virome will be described in detail in section 5 of this review.

There are few studies on the specific role of the urinary microbiota in the maintenance of homeostasis and its underlying mechanisms. Similar to other human microbial communities, the urinary microbiota could play a role in modulating the immune response (Jones-Freeman et al., 2021). In fact, it has been shown that some microbial metabolites may be involved in the regulation of the immune response and inflammation during urinary diseases (El-Deeb et al., 2019).

### Healthy Female Urinary Microbiome

The most abundant genus found in the urinary microbiome in healthy women is *Lactobacillus* (Fouts et al., 2012; Pearce et al., 2014; Modena et al., 2017; Price et al., 2020). However, not all *Lactobacillus* species are associated with a healthy microbiota. In fact, *L. crispatus* has been associated with a state of health, while other species such as *L. gasseri*, have been associated with pathologies, such as urgency urinary incontinence (UUI) (Pearce et al., 2014). In addition, a decrease in the abundance of *Lactobacillus* has been related to pathological states, since its detriment favors the colonization of disease-causing uropathogens (Fouts et al., 2012). *Gardnerella* comes a close second in terms of abundance, with *Gardnerella vaginalis* being the most abundant species harboring some pathogenic strains; some of them might cause urinary tract infections mostly in women, and less frequently in men (Pearce et al., 2014; Ruiz-Gómez et al., 2019).

Urinary bacterial communities are grouped into urotypes in which a particular bacterial genus predominates (Mueller et al., 2017). Various urotypes, named after its respective dominant genus, have been identified, such as *Prevotella, Sneathia, Gardnerella, Atopobium, Lactobacillus, Shigella, Escherichia, Enterococcus, Streptococcusor* and *Citrobacter* (Gottschick et al., 2017). Some of them, such as urotypes dominated by *Lactobacillus crispatus*, *Gardnerella vaginalis* and *Atopobieum vaginae*, are exclusive for healthy women, while others appear frequently associated with some disease.

The origin of the urinary microbial community is not clear, but different hypotheses are being under consideration in this regard. The anatomical proximity between the vagina and the urinary tract suggests that vagina might be the main source of the urinary microbial community. Two recent reports have proposed that the female urinary microbiota and the vaginal microbiota are interconnected. In the first study (Thomas-White et al., 2018a), the authors start analyzing cultured bacteria from the female bladder and through a detailed comparison of the bladder microbiome with the gastrointestinal and vaginal microbiomes, they find a close similarity between the vaginal and bladder microbiota, with distinctive functional abilities compared to those observed in the gastrointestinal microbial communities. A whole metagenome analysis of bacterial strains isolated from vaginal and bladder samples from the same donor revealed a great similarity between strains considering not only emerging uropathogens, such as *Escherichia coli* and *Streptococcus anginosus*, but also commensal members associated with health such as *Lactobacillus iners* and *L. crispatus*. Based on this finding, the author proposes the existence of a single urogenital microbiota in both niches.

In the second study, Komesu et al. (2020) studied the relationship between vaginal and urine microbiome by 16S rRNA gene sequencing in samples from women suffering mixed urinary incontinence and asymptomatic controls. They observed a correlation at the operative taxonomic units (OTUS) level between certain bacteria, such as *Gardnerella, Prevotella, Ureaplasma* or *Lactobacillus*, in urine and vaginal samples, suggesting the existence of a common urogenital microbiota. Despite the common traits, the urinary and vaginal microbiome show some differences, such as the absence of the urinary genera *Tepidomonas* and *Flavobacterium* in the vaginal microbiota.

However, a recent study proposes that the origin of the urinary microbiota is the gut. These authors show that 64% of species identified in urine samples, using culture and 16S rRNA gene sequencing-based methods, overlap with identified species in the gut microbiota, while only 31% overlap with species isolated from the vagina (Dubourg et al., 2020) (**Figure 3B**). Additionally, the reduction of UTI incidence after fecal microbiota transplantation seems to support this hypothesis (Tariq et al., 2017).

### Healthy Male Urinary Microbiome

The number of reports on the male urinary microbiome is significantly less than those on the female urobiome, especially in the case of healthy subjects. The first studies on the male urobiome characterized the microbial community in midstream urine and urethral swab samples in patients with and without sexual transmitted infections. 16S rRNA gene amplification and sequencing made it possible to describe the presence of a male urinary microbial community in healthy individuals, as well as a dysbiosis associated with disease (Nelson et al., 2010; Dong et al., 2011).

A recent study has analyzed the male urinary microbiome in patients with and without urinary tract symptoms using EQUC and 16S rRNA sequencing. This study has shown the importance of using catheterized urine samples in male urobiome studies, as well as in women. However, the small sample size in this study has impaired the identification of significant differences in the microbial communities of both populations (Bajic et al., 2020).

As mentioned previously, the differences found in the composition of the healthy male and female urinary tract microbiome are slight (Gottschick et al., 2017; Modena et al., 2017). Urotypes such as those dominated by *Prevotella, Shigella, Enterococcus, Streptococcus* and *Citrobacter* in the female urinary microbiota have also been found in men (Gottschick et al., 2017). Similar to *Lactobacillus* being the main genus in the female urinary microbiota, the male microbiota is characterized by the predominance of *Corynebacterium* (Fouts et al., 2012) and *Streptococcus* (Modena et al., 2017). *Lactobacillus* and *Pseudomonas* genera have also been described as members of the male microbiota (Moustafa et al., 2018), although in the case of *Lactobacillus* its proportion is lower compared to women (Modena et al., 2017). *Staphylococcus haemolyticus* has been identified as an abundant species in healthy men (Groah et al., 2016). Other shared genera in the male and female urinary microbiota are coagulase-negative staphylococci and *Eubacterium* (Kogan et al., 2015).

### Changes in the Urinary Microbiome Associated With Age

The effect of age on the microbial profiles in the urinary tract has been scarcely analyzed. In general, no age-related differences have been found in the diversity of microbial communities (Curtiss et al., 2018). A reduction in the relative abundance of *Lactobacillus* (Liu et al., 2017; Curtiss et al., 2018; Komesu et al., 2018), *Bifidobacteria, Sneathia, Shuttlewothia* or *Bacillus* (Liu et al., 2017) has been observed in elderly women. Conversely, genera such as *Mobiluncus*, *Oligella* or *Porphyromonas*, have a higher prevalence in postmenopausal women (Curtiss et al., 2018). Moreover, some urotypes are differently distributed according to age, *Escherichia* urotype has been linked to healthy older women, whereas *Gardnerella* urotype has been associated with young women (Price et al., 2020).

In addition to changes in relative abundance, four genera have been exclusively identified in men and women over 70 years: *Jonquetella, Parvimonas, Proteiniphilum* and *Saccharofermentans* (Lewis et al., 2013). The exclusive presence of *Parvimonas* in postmenopausal women has been recently confirmed (Curtiss et al., 2018). However, due to the low number of participants in Lewis’ study (6 men and 10 women), the exclusive presence of the other three genera in the elderly microbiota still needs to be confirmed.

## Influence of the urinary microbiome on disease

The growing relevance of the study of the urinary microbiome stems from the fact that alterations in its composition have been associated with the development of different diseases, such as urinary tract infection, renal carcinoma, urgency urinary incontinence, overactive bladder, among others. Next, we will briefly describe what has been reported on this aspect to date.

### Urinary Tract Infection

Urinary tract infection (UTI) is one of the most acquired bacterial infection in humans. Most cases occur as acute uncomplicated infections in healthy individuals. UTI is characterized by an initial colonization of the urethral cavity and a subsequent infection of the lower urinary tract up to the bladder, causing urethritis and cystitis respectively (Hooton, 2012). Some infections reach the kidneys causing pyelonephritis and even spread through the bloodstream, leading to a systemic infection (urosepsis) (Lee and Neild, 2007). UTI has been commonly associated with *Escherichia coli* (80% of the cases) but other commensal members of the gut microbiota, such as *Enterococcus*, and *Staphylococcus*, are also involved (Khasriya et al., 2013). Interestingly, there seems to be a correlation between an increase in the intestinal abundance of these genera and a higher prevalence of UTI (Paalanne et al., 2018; Magruder et al., 2019).

A recent study has described the presence of *Escherichia coli* in patients suffering UTI and other urinary disorders, as well as in healthy individuals. Whole genome sequencing has shown no differences in the genomic content of virulence factors genes between strains isolated from patients and healthy individuals. Only some differences in motility genes between isolates from UTI patients and healthy individuals were found. Therefore, *E. coli* is part of the commensal urinary microbiota and other aspects may be determining its involvement in the development of urinary tract symptoms (Garretto et al., 2020). Moreover, *Escherichia coli* has a greater pathogenicity in polymicrobial infections, mainly when it is isolated together with *Enterococcus*, although the mechanisms underlying this coinfection are not well understood (Lavigne et al., 2008; Croxall et al., 2011). Along this same line, it has been described that *Enterococcus faecalis* can modulate its local environment through the emission of signals to promote the growth of other coinfecting organisms. Specifically, *E. faecalis* stimulates the growth and survival of *Escherichia coli* biofilm through the secretion of L-ornithine, used by *Escherichia coli* to synthesize the enterobacterium siderophore under iron limiting conditions, which would otherwise impede its growth (Keogh et al., 2016).

In addition, different bacteria from the urinary commensal microbiota, such as *Corynebacterium glucuronolyticum, Streptococcus gallolyticus, Aerococcus sanguinicola*, have been described as opportunistic uropathogens causing infections (Jiménez-Guerra et al., 2018; Pereira-Pérez et al., 2019; Ruiz-Pino et al., 2019).

Similarly, the vaginal microbiota might influence host susceptibility to UTI. Women with recurrent UTI become more resistant if their vaginal microbiota is modified by the administration of probiotics, specifically *Lactobacillus crispatus* (Stapleton et al., 2011). Moreover, women with bacterial vaginosis caused by overgrowth of anaerobic species, such us some strains belonging to *Gardnerella vaginalis*, suffer more UTI than women with healthy microbial communities, composed mainly of *Lactobacillus* (Sumati and Saritha, 2009). It has been shown that temporary exposures to some strains of *Gardnerella vaginalis* triggers the activation of *Escherichia coli* from dormant intracellular reservoirs in the bladder, enhancing the chance of developing recurrent UTI through the induction of apoptosis and interleukin 1-receptor-mediated injury in bladder epithelial cells (Gilbert et al., 2017). In summary, these results extend the classic concept of the pathogenesis of UTI, suggesting that the disease may be driven by occasional exposures of the urinary tract to gut or vagina-associated bacteria, not traditionally considered as uropathogenic.

In the last few years, the use of NGS focused on 16S rRNA gene has revealed that UTI may have a polymicrobial origin involving the implication of certain bacteria such as *Actinobaculum schaalii* and *Aerococcus urinae*, which might be overlooked by standard techniques (Domann et al., 2003; Imirzalioglu et al., 2008; Gomez et al., 2011). This finding has led to the consideration that some cases of UTI may result from an imbalance in the urinary microbiota repertoire rather than an invasion by an exogenous pathogenic organism.

Archaea have been recently reported as members of the urobiome. Using specific protocols to sequence the archaeal 16S rRNA and culture media for archaeal methanogens *Methanobrevibacter smithii* was detected along enterobacteria in urine samples from patients suffering UTI (Grine et al., 2019). It has been proposed that this archaeon could trigger a dysbiosis in the urinary microbiota favoring the growth of uropathogens and, therefore, the development of UTI. However, this hypothesis requires additional investigation. Methanogens produce methane as byproduct under anaerobic conditions, being able to use it to methylate other molecules, such as heavy metals, and thus producing toxic metabolites for human and bacterial cells. Methanogens have been previously identified as part of the microbiota in the gut (Grine et al., 2017; Nkamga et al., 2017), the oral cavity (Nguyen-Hieu et al., 2013) and the skin (Probst et al., 2013; Moissl-Eichinger et al., 2017). However, their role as pathogens has not yet been well established. Although there is evidence to suggest that some Archaea are emerging pathogens, further research is required to gain insight into the methanogen’s repertoire in the human urinary tract and to understand its possible implication in infections and other diseases.

**Figure 4:**
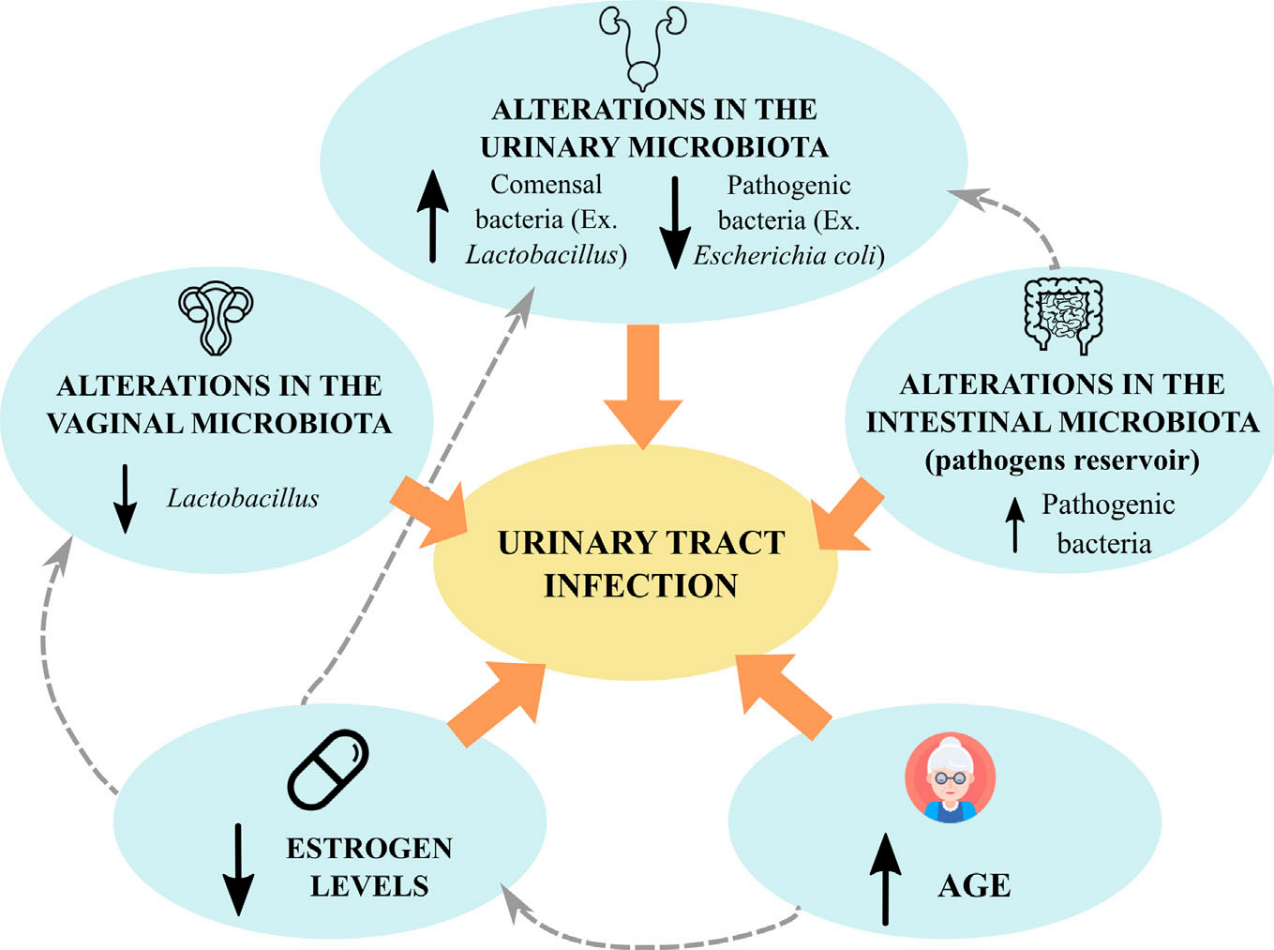
Risks factors for urinary tract infections related to the microbiota. UTIs may be influenced by different risk factors related to microbial communities: alterations in the urinary microbiota (either an increase in uropathogens or a decrease in commensal bacteria) (Thomas-White et al., 2018a), alterations in the intestinal microbiota (increase in uropathogens in the intestinal reservoir) (Magruder et al., 2019) and increasing age, which is related to a decrease in estrogen levels in women and fluctuations in abundance of *Lactobacillus* (Thomas-White et al., 2018a).

One of the main problems for urinary tract surgery is the appearance of postoperative UTI. The predisposition to suffer this infection is associated with a decrease in some *Lactobacillus* species, such as *L. iners*, as well as an increase in uropathogens such as *Escherichia coli, Klebsiella pneumoniae* or *Pseudomonas aeruginosa*. This alteration in the urinary microbiota, along with other risk factors, such as age or a decrease in estrogen levels, determine the appearance of postoperative UTI (**Figure 4**) (Raz, 2011; Thomas-White et al., 2018a).

Different fungal species have been identified in the urine of individuals with urinary tract symptoms such as *Clavispora lusitaniae, Lodderomyces elongisporus, Meyerozyma guilliermondii* and *Malassezia globose*, as well as different species belonging to *Candida* genus, such as *C. albicans, C. orthopsilosis, C. tropicalis, C. glabrata, C. lusitaniae*, among others (Heras-Cañas et al., 2015; Moustafa et al., 2018).

Microbial identification based on molecular techniques has allowed a more accurate diagnosis of UTI, detecting bacteria and other uncultured uropathogenic microorganisms and avoiding false negative results. In this regard, a better treatment choice is achieved in patients with urinary tract infection (Ishihara et al., 2020).

### Cancer of the Urinary Tract

Urothelial carcinoma, also known as transitional cell carcinoma (TCC), is the most common type of bladder cancer. One of the risk factors for its development is chronic UTI (Akhtar et al., 2018). Since the microbiota is associated with the development of cancer in various tissues, it is possible to speculate that it could also be involved in TCC. However, to date very few studies have explored the role of microbiome in urologic malignancy, and most of them with small sample size.

One study compared the microbiome in urine from 31 TCC patients and 18 healthy controls, finding in TCC patients a higher abundance of *Acinetobacter, Anaerococcus*, *Sphingobacterium* and members of *Sphingobacteriaceae* family; pathogenic bacteria such as *Herbaspirillum, Porphyrobacter* and *Bacteroides* were also increased in TCC patients, as well as urinary infections caused by *Staphylococcus*. The authors suggest that microbiome-mediated modifications in extracellular matrix and its consequent inflammation can play a role in the carcinogenesis (Wu et al., 2018). A second study analyzed the microbial composition of urine in 12 TCC patients and 11 controls, noticing an enrichment in *Fusobacterium* genera in the TCC group, which has also been associated with colorectal cancer (Bučević Popović et al., 2018). Finally, a third report studied the urinary microbiome from 29 TCC patients and 26 non-cancer patients and found higher abundance of *Actinomyces europaeu* in patients suffering from bladder cancer, which suggests this species may be an indicator for this disease (Bi et al., 2019).

Recently, one study has analyzed the implication of the urobiome in prostate cancer comparing the urinary microbiome in men with positive versus negative diagnosis for the disease. The study concludes that the presence of pro-inflammatory or pathogenic bacteria, and those involved in urinary tract pathologies, such as UTI or prostatitis, favors the development of prostate cancer (Shrestha et al., 2018). Some of these bacteria are *Actinobaculum schaalii, Anaerococcus lactolyticus, Varibaculum cambriense, Propionimicrobium lymphophilum* and *Ureaplasma* species.

All these studies suggest a role of the urinary microbiota in the development of bladder cancer. However, larger-scale studies with higher number of patients and, if possible, urine samples obtained by bladder catheterization will be essential to clarify the role of urobiome dysbiosis in cancer of the urinary tract.

### Urinary Incontinence

Urinary incontinence (UI) is a disorder of the urinary tract characterized by uncontrolled loss of urine, appearing more frequently in women. Three types of urinary incontinence have been described: stress urinary incontinence (SUI), in which urine loss is associated with cough or physical exertion; urgency urinary incontinence (UUI), associated with an uncontrollable desire to urinate; and mixed urinary incontinence (MUI), a combination of the aforementioned two (Haylen et al., 2010; Govender et al., 2019). Despite the fact that some risk factors have been identified, such as age, body mass index, parity or hormones, there is still a gap in our knowledge when it comes to UI (Aoki et al., 2017). However, studies based on 16S amplicon sequencing have established an association between the urinary microbiota and the development of UI.

One study compared the urobiome in catheterized urine samples from 60 female patients suffering UUI and 58 healthy women using sequencing-based methods and the EQUC technique. UUI patients showed a lower abundance of *Lactobacillus* and a higher abundance of *Gardnerella*, along with other genera such as *Actinobaculum, Actinomyces, Aerococcus, Arthrobacter, Corynebacterium, Oligella, Staphylococcus and Streptococcus* (Pearce et al., 2014). Although *Gardnerella* has been described as a member of the commensal urinary microbiota, some *Gardnerella* strains might be involved in certain pathologies such as bacterial vaginosis, allowing the differentiation between pathogenic and commensal *Gardnerella vaginalis* strains (Harwich et al., 2010). Additionally, differences in *Lactobacillus* species have also been observed between patients and controls, with an increased abundance of *L. gasseri* in UUI patients and of *L. crispatus* in healthy individuals (Pearce et al., 2014).

Interestingly, a recent study has shown that uropathogenic bacteria in the urobiome of patients suffering UUI, such as *Escherichia coli* or some *Gardnerella vaginalis* strains, can induce the release of ATP and a Ca^2+^ influx into uroepithelial cells, ultimately favoring the contraction of the smooth muscle cells in the urinary bladder. On the other hand, *Lactobacillus* members impair the production of ATP and the Ca^2+^ flow generated by these bacteria, preventing muscle cells contraction (Abbasian et al., 2019). Therefore, members of the urinary microbiota might be involved in the contraction of the urinary bladder contributing to the pathophysiology of UUI.

A second study also analyzed catheterized urine samples from 10 patients suffering UUI and 10 healthy individuals by sequencing-based methods and identified certain enriched genera in UUI patients, such as *Methylobacterium, Brevundimonas, Chitinophaga, Sphingomonadales*, among others (Karstens et al., 2016). In addition, authors also described a reduction in *Prevotella, Comamonadaceae, Nocardioides* and *Mycobacterium* in UUI patients. In general, 14 bacterial genera showing differences in abundance between patients and controls were identified. Nevertheless, some of the most abundant genera identified by Pearce et al. (2014) (Pearce et al., 2014) were not found in this study, which indicates that additional studies are needed to confirm these results.

To date, few studies have been conducted regarding the other types of urinary incontinence. One study compared the urobiome in catheterized urine samples from 123 women with MUI and 86 healthy controls. Patients with MUI showed a decreased relative abundance of *Lactobacillus* and an increase in *Gardenerella* and *Prevotella*, similar to what had been previously described in UUI patients (Komesu et al., 2018). In contrast, a study analyzed the microbial community in midstream urine samples and some catheterized urine samples from patients with SUI and found no differences between patients and healthy individuals (Thomas-White et al., 2017). These results may be skewed due to the use of midstream urine samples in the SUI-associated urobiome study, which may contain microbial contamination. However, it is also possible that the differences in the urinary microbiome observed in MUI patients are due to the UUI-associated component of the disease.

### Kidney Transplant Dysfunction

One of the main problems in kidney transplantation is the rejection of the transplant by the recipient and the consequent dysfunction of the transplanted organ. Interstitial fibrosis and tubular atrophy (IFTA) are a pathological process triggered by an excessive accumulation of extracellular matrix and its interaction with inflammatory cytokines. This injury has been associated with a reduced survival of the transplanted organ (Nankivell et al., 2001; Li and Zhuang, 2014).

The relationship between the urinary microbiota and the development of IFTA was analyzed in a study comparing midstream urine samples from 25 kidney transplanted patients with IFTA evidences, 23 patients with normal transplant survival and 20 healthy controls. Using high-throughput 16S sequencing, a lower abundance of *Lactobacillus*, in the case of women, and a lower abundance of *Streptococcus*, in the case of men, were detected in patients who showed histological damage associated with IFTA. Accordingly, an increase in the abundance of pathogenic bacteria, such as *Propionibacterium acne, Prevotella disiens, Gardnerella vaginalis* or *Finegoldia magna*, among others, was also found. It is possible that the enrichment in uropathogens might favor the development of an enhanced immune response, increasing the chances of transplant rejection (Modena et al., 2017).

Another study compared the urobiome in kidney transplanted patients who showed allographic dysfunction and patients with normal transplant survival. A greater abundance of *Corynebacterium* was found in patients with transplant dysfunction, as well as differences in the abundance of various genera such as *Rhodococcus, Facklamia, Streptococcus* or *Fusobacterium*, among others (Wu et al., 2018). However, it is not clear whether these urinary microbiota alterations are the cause of transplant dysfunction or its consequence. Additional research on the dysbiosis in the urinary bacterial community and its association with allographic dysfunction may allow its early diagnosis.

### Other Disorders

Other urinary disorders have been associated with urobiome dysbiosis. Overactive bladder syndrome (OAB) is a disease characterized by a constant need to urinate with similar symptoms as those described for urinary incontinence (Dhaliwal and Wagg, 2016; Govender et al., 2019). One study compared the urinary microbiota in midstream urine samples from 63 female patients suffering OAB and 35 healthy controls. Patients showed a lower abundance of *Lactobacillus* and a higher abundance of *Proteus* compared to healthy individuals (Curtiss et al., 2017). These results were supported by a second study in which the urinary microbiome of 30 OAB patients and 25 healthy individuals was compared using catheterized urine samples (Wu et al., 2017). A third study analyzed the urobiome in catheterized urine samples from 126 women about to undergo urinary surgery. Approximately half of the patients showed OAB symptoms, being this symptomatology related to an increased abundance of *Atopobium vaginae* and *Finegoldia magna.* However, the presence of *Atopobium vaginae* in patients with these symptoms, as well as in healthy women, seems to indicate the existence of pathogenic and commensal strains depending on the surrounding environment (Fok et al., 2018).

Neuropathic bladder is a urinary disfunction derived from alterations in the central nervous system, which impair correct urine storage and emptying of the urinary bladder. This disorder might favor the appearance of other pathologies such as UTI or renal dysfunction (Roth and Cain, 2018). A relationship between the urinary microbiota and the presence of neuropathic bladder has been verified in a study comparing the urobiome from 24 patients and 23 healthy controls. The urinary microbiota in patients was characterized by an increased abundance of pathogens, such as *Escherichia coli, Enterococcus faecalis, Pseudomonas aeruginosa, Klebsiella pneumoniae* and members of the *Actinobaculum* genus, such as *A. massiliense, A. schaalii, A. suis* and *A. urinale* (Groah et al., 2016).

Interstitial cystitis/bladder pain syndrome is characterized by pain and discomfort in the bladder and lower urinary tract, with absence of other pathologies such as UTI (Hanno et al., 2011). One study analyzed the urinary microbiome in midstream urine from 181 female patients with interstitial cystitis/bladder pain syndrome and 182 healthy female controls using the PLEX-ID molecular diagnostic platform, which combines PCR amplification with mass spectrometry. The authors observed differences in the abundance of certain bacteria, such as an increase in *Lactobacillus gasseri* or a reduction in *Corynebacterium* and identified exclusive bacteria in patients, such as *Proteus mirabilis, Pseudomonas aeruginosa, Francisella tularensis, Mycoplasma hyorhinis, Helicobacter hepaticus, Clostridium perfringens*, among others (Nickel et al., 2019). A similar study demonstrated a higher prevalence of *Candida* and *Saccharomyces* in the urobiome of patients with signs of interstitial cystitis/bladder pain syndrome, thus being possibly related to the development of this pathology (Nickel et al., 2016). However, other recent studies using 16S rRNA gene sequencing have not identified changes in the urinary microbiota related to this syndrome (Bresler et al., 2019; Meriwether et al., 2019). Therefore, more studies are required to reach a consensus on both positions.

On the other hand, chronic prostatitis/chronic pelvic syndrome is characterized by similar symptoms as those described for interstitial cystitis/bladder pain syndrome, appearing in this case only in men (Polackwich and Shoskes, 2016). This syndrome has also been linked to dysbiosis of the urinary microbiota. A study comparing the urobiome in midstream urine samples from 25 male patients and 25 male healthy controls showed an increase in the abundance of anaerobic bacteria such as *Clostridia, Bacteroides* or *Porphyromonas*, as well as a decrease in other genera such as *Bacilli.* In addition, functional analysis of this community showed an increase in sporulation pathways, which may be attributed to resistance mechanisms implemented by *Clostridia* (Shoskes et al., 2016).

Nephrolithiasis is a common urological disease characterized by the presence of calcium-based kidney stones (Wang W. et al., 2017). This pathological condition has been associated with an alteration of the urinary microbiota. A recent study comparing the bacterial community in catheterized urine samples from 22 male patients and 21 male healthy controls described a reduction in species diversity and an alteration in the microbial community in patients. *Acinetobacter* was overrepresented and *Prevotella* was reduced in male patients. *Prevotella* has been described as a protective bacterium against inflammation through the production of short-chain fatty acids; therefore, its reduction promotes a proinflammatory state and formation of kidney stones (Xie et al., 2020). In addition, a second study has described the relationship between the urinary microbiota and the development of hypertension associated with nephrolithiasis. A link between changes in blood pressure and alterations in the urinary microbial community was established in 50 catheterized patients with kidney stones and different stages of hypertension. An increase in microbial nitrogen and nucleotide metabolism and a decrease in adherens junction pathways have been also described (Liu et al., 2020).

Liu et al. (2017) identified a relationship between type 2 diabetes mellitus, which has a higher prevalence in older individuals, and urinary microbiota. Elderly individuals suffering this pathology showed a lower abundance of some phyla, such as Nitrospirae, Verrucomicrobia and Planctomycetes, as well as a lower abundance of *Lactobacillus* genus, although a greater abundance of *L. iners* was observed in comparison with healthy controls. In addition, a recent study analyzed the urobiome of 32 patients suffering type 2 diabetes mellitus and 26 healthy controls and identified an increase in the relative abundance of 10 genera associated with type 2 diabetes, such as *Escherichia, Shigella, Klebsiella, Enterococcus* and *Aerococcus*, among others, having many of them been described as pathogenic bacteria (Chen et al., 2019).

Therefore, many urinary pathologies are related to urobiome dysbiosis and its analysis might be a key factor to consider when designing preventive, diagnostic or therapeutic strategies.

## Urinary Virome

The term microbiota includes not only bacteria or even fungi, but also both human and bacteriophage viruses. The set of all viral genetic material in a certain biological niche is known as virome.

A metagenomic study of whole metagenome shot-gun sequencing from 49 midstream urine samples has allowed the identification of different human viruses such as human papillomavirus, molluscum contagiosum virus, BK and JC polyomavirus, herpesvirus and anellovirus (Moustafa et al., 2018). BK polyomavirus has been associated with urinary tract infections in immunocompromised patients (Paduch, 2007). However, other human viruses, such as human papillomavirus, have been identified in patients and in healthy individuals (Santiago-Rodriguez et al., 2015). JC polyomavirus has been identified by whole metagenome sequencing in catheterized urine samples from patients with overactive bladder (Garretto et al., 2018). The presence of viruses in the urine of patients could open up a new field of study for the analysis of new mechanisms for the local transmission of infections, as well as for the design of innovative preventive measures and new therapeutic strategies. However, knowledge on the role of human viruses in the urinary tract is scarce and requires further investigation.

Nevertheless, the urinary virome is mainly formed by bacteriophages, viruses which infect urinary bacteria such as *Staphylococcus, Escherichia coli* or *Enterococcus* (Santiago-Rodriguez et al., 2015; Garretto et al., 2018; Moustafa et al., 2018). Many lytic bacteriophages have been identified in the urinary tract, like φCTX-like *Pseudomonas aeruginosa*-infecting phage isolated from kidney stones (Johnson et al., 2019), or *Escherichia coli*-infecting phages isolated from the bladder of women suffering UUI (Malki et al., 2016). Lysogenic phages have been also detected in the urinary bladder from both healthy individuals and patients with urinary tract symptoms (Miller-Ensminger et al., 2018). A thorough review about bacteriophages in the urinary tract has been recently published (Garretto et al., 2019), although, for the most part, the role of bacteriophages in the urinary microbiota remains unknown. These phages could have an important role in the maintenance of health due to their ability to infect uropathogenic bacteria in the urinary microbiota.

## Urobiome Research and New Therapies

The recent characterization of the urinary microbiome and its relationship with disease has led to the development of urobiome-targeted therapies.

UTI is usually treated with antibiotics and in consequence, the importance of an accurate bacterial identification has become more relevant due to the prevalence of broad-spectrum antibiotic- resistant uropathogens (Cueto et al., 2017). In an effort to overcome these resistances, it is imperative to use effective drugs targeted at each specific causative agent. It has been observed that treatment response in different UTIs varies depending on the causative pathogen. For instance, cystitis caused by *Aerococcus urinae* can be effectively treated with Nitrofurantoin, while pyelonephritis caused by this same pathogen can be treated with Ciprofloxacin; however, these antibiotics are ineffective against infections caused by *Aerococcus sanguinicola*, a different species belonging to the same genus, (Oskooi et al., 2018). The recent identification of a methanogenic archaea causing UTI has shown that this uropathogen is resistant to a wide range of antibiotics commonly used to treat urinary infections such as Fosfomycin, Cotrimoxazole (Sulfomethoxazole and Trimethoprim), Amoxicillin-Clavulanate and Ofloxacin (Grine et al., 2019). In addition, some pathogens, such as *Enterococcus faecium*, are resistant to commonly used beta-lactams. The implementation of non-specific antibiotics in infections is not only ineffective, but it also causes proliferation of pathogens in the intestinal microbiota due to the depletion of commensal bacteria, favoring the appearance of urinary tract infections (Magruder et al., 2019).

Another contributing factor to therapeutic failure is the fact that some pathogens reside inside cells and are therefore inaccessible to the majority of drugs. Small concentrations of antibiotics might access cell cytoplasm, and it has been observed that in the case of Ciprofloxacin, it triggers the release of adenosine triphosphate (ATP) by intracellular *Escherichica coli* with its consequent increase in Ca^2+^ flow, thus promoting contractility in the urinary bladder and the appearance of pathologies such as urgency urinary incontinence (Abbasian et al., 2019).

Additionally, it has been widely studied that the antibiotics used against UTI might affect the gut microbiota, causing a reduction in abundance and diversity and, therefore, intestinal dysbiosis (Elvers et al., 2020). Similarly, the exposure of the urinary microbiota to antibiotics might generate an alteration in the urinary microbial community. A study has described that the use of antimicrobial drugs is associated with a dysbiosis of the urogenital microbiome, although further research is required to confirm that such dysbiosis is the consequence and not the cause that initially prompted the treatment (Mulder et al., 2019).

In addition, microbial diversity also determines treatment response. A study showed that patients suffering urgency urinary incontinence with a lower diversity in the urinary microbiota had less serious symptomatology and a greater treatment response to Solifenacin, compared to those with a greater microbial diversity (Thomas-White et al., 2016).

Taking all this into consideration, the development of new therapeutic tools regarding urinary tract disorders is becoming increasingly relevant. The use of probiotics is a key aspect in this regard; it has been seen that the intravaginal administration of probiotic *Lactin-V*, composed of *Lactobacillus crispatus*, enables long-term colonization of the urinary microbiota by commensal bacteria and a lower incidence of UTI (Stapleton et al., 2011). In addition, it has been shown that the oral administration of other species belonging to *Lactobacillus* genu*s*, such as *L. acidophilus* and *L. plantarum*, along with vitamin A and cranberry extract, reduces the appearance of UTI (Koradia et al., 2019). The use of cranberry extract has been shown to inhibit *Escherichia coli* adhesins, making it difficult to colonize the urinary bladder (Howell et al., 2010).

Probiotics can be orally or vaginally administered, although administration by bladder instillation has been found to be advantageous over the previous ones since it allows direct colonization of the urinary bladder. This colonization leads to a shift in bacterial proportions towards a healthy microbial composition with positive effects against UTIs (Forster et al., 2021).

Fecal microbiota transplantation is a recently developed therapy which is being under consideration against urinary tract infections. These transplants induce an increase in the abundance of *Lactobacillus* and a decrease in the abundance of *Enterobacteriaceae* family (Biehl et al., 2018). However, only one patient was analyzed in this study; a higher number of subjects would be required to confirm these results. Similarly, in another study, fecal microbiota transplantation caused a reduction of UTI incidence and an increased susceptibility of uropathogenic microorganisms to antibiotics (Tariq et al., 2017).

Finally, considerable effort is being put in the study of lysogenic bacteriophages (Miller-Ensminger et al., 2018) as therapeutic tools for urinary tract infections since they were first identified. One study has shown that three bacteriophages belonging to *Syphoviridae, Myoviridae* and *Podoviridae* subfamilies reduced the levels of UTI-causing *Escherichia coli*, including antibiotic resistant strains, both in *in vitro* and *in vivo* murine models (Galtier et al., 2016). A second study analyzed the effect of bacteriophages administration in patients with a positive culture for uropathogens. A solution containing a mixture of active bacteriophages against *Staphylococcus aureus, Escherichia coli, Streptococcus* spp.*, Pseudomonas aeruginosa* and *Proteus spp* (Pyo bacteriophage preparation) was administered to nine patients by intravesical instillation. The results derived from this study showed that bacteriophage administration safely reduced, with no side effects, levels of *Escherichia, Enterococcus, Pseudomonas* and *Streptococcus*, which can act as urinary tract pathogens (Ujmajuridze et al., 2018). Therefore, bacteriophages can be used as a possible secure therapy against uropathogens and as an effective therapy against antibiotic-resistant bacteria. However, more studies are required to understand the role of the bacteriophages in the Urinary Tract Health.

## Future Perspectives of Urobiome Research

Given the initial consideration of sterility regarding the urinary bladder, the study of the microbial community in the urinary tract has made considerable progress. However, there is still a long way ahead. Firstly, more studies are needed for the optimization of the techniques used for microbial identification and for the establishment of a core microbial community in healthy individuals. More studies are also needed to analyze urinary microbial profiles and their association with different urinary tract pathologies, allowing its use as diagnosis, prognosis and treatment biomarkers. Secondly, more studies regarding the relationship of the intestinal microbiota and the urobiome are required, including its implication in the development of urinary infections. These types of studies may shed light on prevention and treatment strategies for UTIs, especially those targeting the intestinal microbial community, which is known to be a reservoir for uropathogens (Bahadori et al., 2019; Thänert et al., 2019). In particular, gaining insight into the mechanisms underlying interactions between the intestinal and urinary microbiota might be of great interest for the development of gut microbiome-targeted therapies for UTIs.

Finally, research on uropathogens physiopathology and their pathogenicity, as well as on the mode of action of probiotics and its effectiveness in different scenarios and combinations with other compounds, might contribute to the development of new additional therapies apart from antibiotics. Although new therapeutic tools based on the administration of bacteriophages are in early stages of development, their study and application are gaining significance.

## Author Contributions

VP-C and JG-S wrote de manuscript. VP-C performed the figures. All authors contributed to the article and approved the submitted version.

## Funding

This study was supported by “Programa Estatal de Investigación, Desarrollo e Innovación Orientada a los Retos de la Sociedad” (grant SAF-SAF2015-71714-RMINECO/FEDER) and by “Instituto de Salud Carlos III” under the frame of EuroNanoMed III (AC18/00008). VP-C was supported by “Programa de Promoción de Empleo Joven e Implantación de la Garantía Juvenil en I+D+i”, MIMECO, Spain, and AS-L was supported by a fellowship from the Ministry of Education, Culture and Sport (FPU 17/05413).

## Conflict of Interest

The authors declare that the research was conducted in the absence of any commercial or financial relationships that could be construed as a potential conflict of interest.
